# Primary Adenocarcinoma of the Urinary Bladder: A Case Report on a Rare Malignancy

**DOI:** 10.7759/cureus.66269

**Published:** 2024-08-06

**Authors:** Prajakta Ghewade, Samarth Shukla, Sunita Vagha, Shivali S Kalode, Pravin Gadkari

**Affiliations:** 1 Department of Pathology, Jawaharlal Nehru Medical College, Datta Meghe Institute of Higher Education and Research (Deemed to be University), Wardha, IND

**Keywords:** neoplasm, primary, enteric type, urachal, malignancy, urinary bladder, adenocarcinoma

## Abstract

Of all primary bladder cancers, primary adenocarcinoma is an uncommon tumor. When considering all tumor origin areas, secondary bladder involvement from carcinoma, whether by direct extension or metastasis, is actually more prevalent than primary adenocarcinoma, despite its rarity. The most common source of subsequent bladder tumors is endometrial, lung, colon, prostate, breast, or other organ adenocarcinomas. Primary bladder adenocarcinoma is thought to result from urothelial metaplasia, which is frequently linked to persistent irritation or inflammation. Bladder exstrophy, recurrent urinary tract infections, long-term irritation from calculi or foreign bodies, and history of schistosomiasis are risk factors. A portion of these malignancies are associated with urachal remnants, where the tumor originates at the dome of bladder. Here we present a case of primary adenocarcinoma in a 44-year-old female patient that originated from the dome of urinary bladder.

## Introduction

Primary bladder adenocarcinoma is the term for a malignant tumor that arises from the urothelium of the bladder and shows histologically pure glandular differentiation. Of all bladder neoplasms, 0.5%-2.5% are primary adenocarcinomas, an extremely rare malignancy [1,2]. With a mean age of 63 years, this tumor is more prevalent in males than in females [3]. Although rare, secondary bladder involvement from carcinoma, either by direct extension or metastasis, is actually more common than primary adenocarcinoma when all tumor-origin regions are considered. Adenocarcinomas of the colon, prostate, breast, endometrial, lung, or other organs are the most common causes of secondary bladder cancers [1,4]. Because benign and malignant glandular tumors can occur in the bladder, accurately diagnosing adenocarcinoma can be difficult [5,6]. Differential diagnosis for a urinary bladder lesion with glandular differentiation may include benign entities, possible precursor lesions, primary tumors, and secondary malignancies. Precursor lesions in primary bladder adenocarcinoma are less developed than in other organs, including the colon and breast. However, it is thought that a number of glandular anomalies, such as tubular adenoma, urachal remnants, and intestinal metaplasia, are the precursors of primary bladder adenocarcinoma [7,8].

## Case presentation

**Figure 1:**
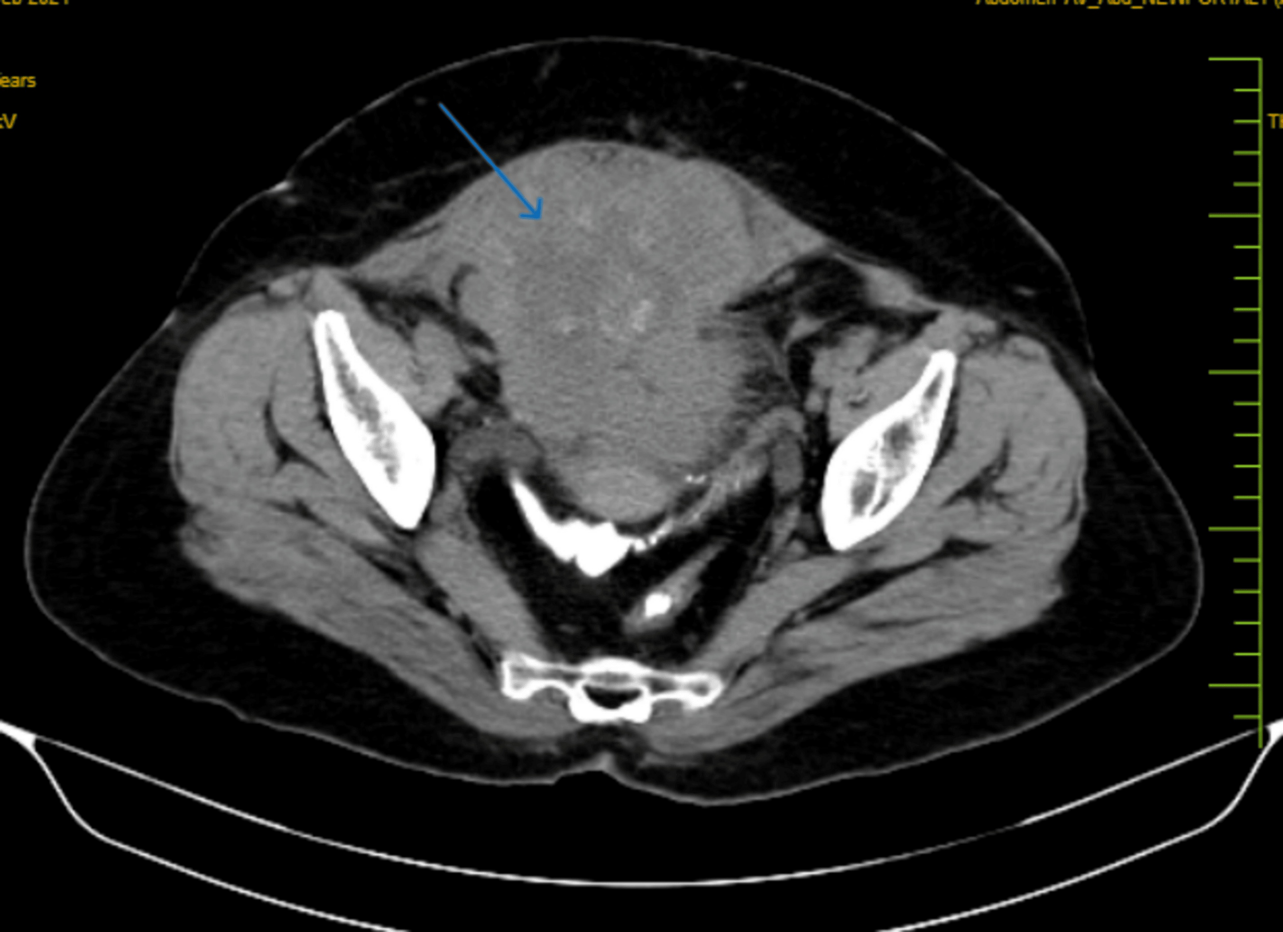
CT scan showing a heterogeneously enhancing lobulated soft tissue mass lesion (blue arrow) CT: computed tomography

A 44-year-old female patient arrived at the hospital complaining of burning micturition for six to eight months, decreased frequency of urination, and hematuria. Additionally, there was a history of decreased appetite and urine incontinence. The preliminary workup was completed consisting of routine blood investigations CBC, liver function test, kidney function test, urine routine microscopy, and imaging studies. A heterogeneously enhancing lobulated soft tissue mass lesion spanning approximately 8.1 x 7.4 x 5.7 cm was seen on a computed tomography (CT) scan along the fundic region of the bladder, accompanied by minimal surrounding fat stranding (Figure 1).

**Figure 2:**
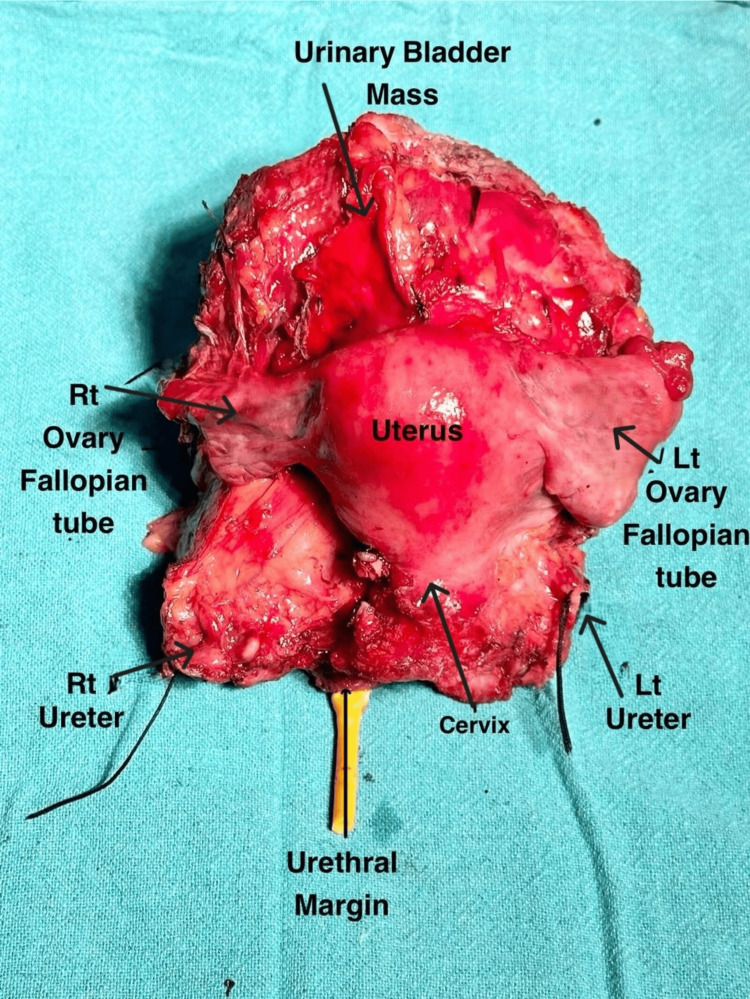
Gross image of the specimen (black arrows) Rt: right; Lt: left

The bladder tumor was removed transurethrally, and the specimen was transferred to a private laboratory. The report raised the possibility of high-grade urothelial carcinoma. The patient received four cycles of neoadjuvant chemotherapy; however, there is no record of this treatment. After one year, she underwent total cystectomy and total abdominal hysterectomy with bilateral salpingo-oophorectomy (anterior exenteration) with ileal conduit creation. The sample was sent to the lab for histopathological evaluation (Figure 2).

**Figure 3:**
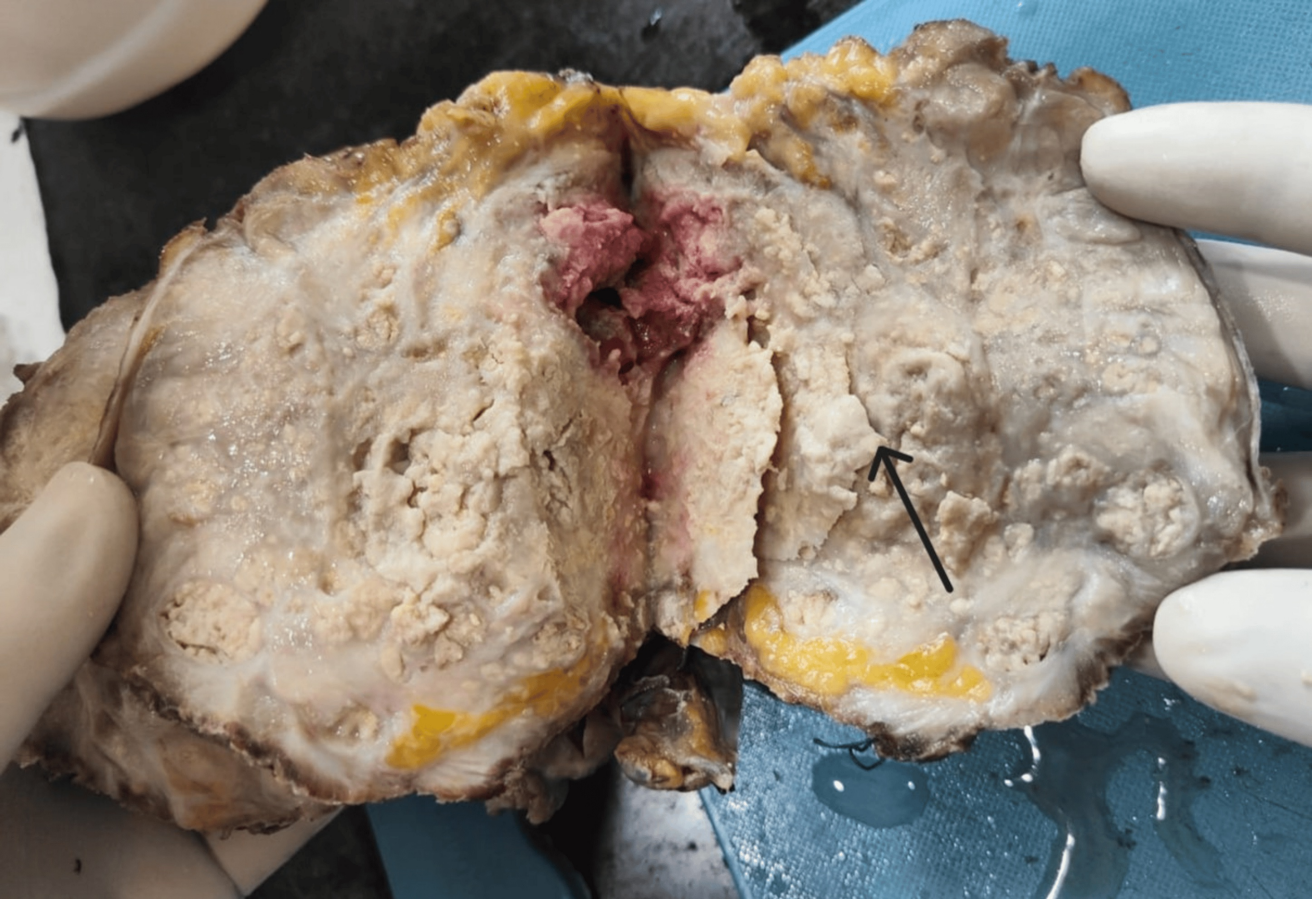
Tumor mass seen on the cut section of the urinary bladder (black arrow)

A cut section of the urinary bladder showed a friable, cheesy ulceroproliferative tumor measuring 10.8 x 9 x 8 cm. The tumor filled the bladder cavity, arising from the dome and infiltrating the bladder wall. The tumor did not involve the uterus, cervix, bilateral ovaries, or fallopian tubes (Figure 3).

**Figure 4:**
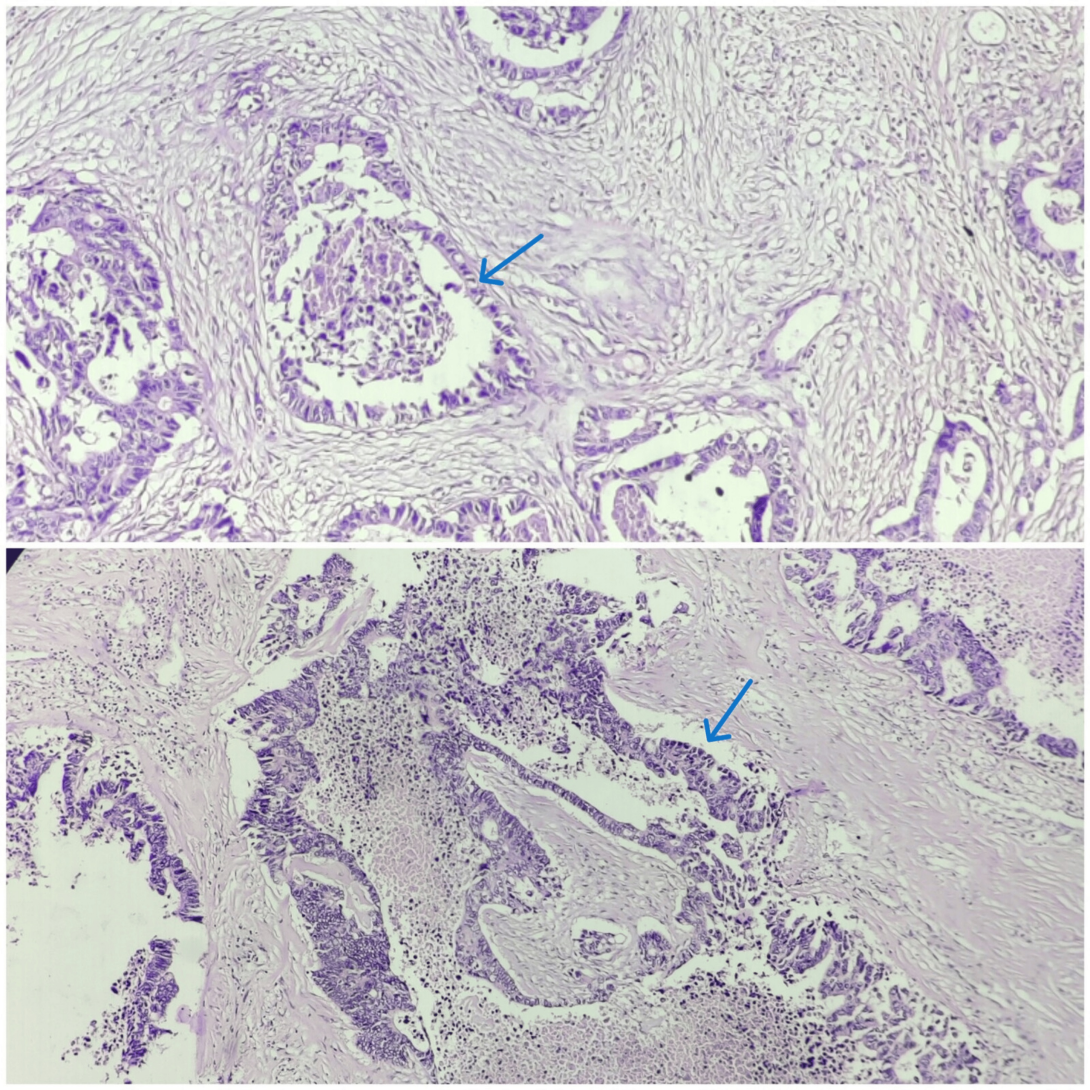
Neoplastic glands lined by pleomorphic, mucin-producing, pseudostratified columnar epithelium (10×; blue arrows)

**Figure 5:**
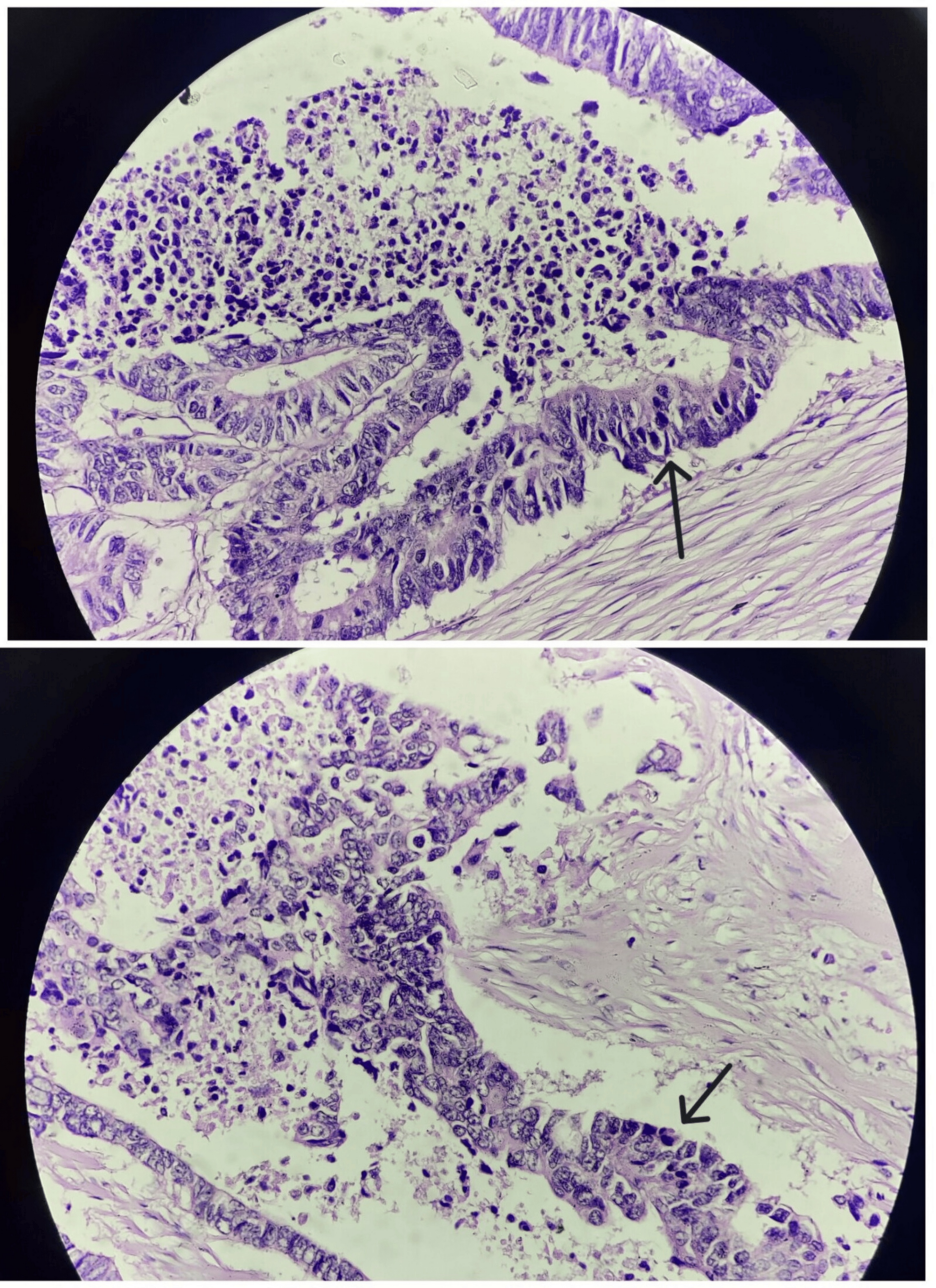
Neoplastic glands lined by pleomorphic, mucin-producing, pseudostratified columnar epithelium (40×; black arrows)

The microscopic picture showed neoplastic glands lined by pleomorphic, mucin-producing, pseudostratified columnar epithelium (Figures 4, 5). It bore similarities to a colonic adenocarcinoma, featuring extensive areas of central necrosis. The histopathological features indicate moderately differentiated adenocarcinoma of the enteric type with invasion up to the muscularis propria. The diagnosis was confirmed by immunohistochemistry, where tumor cells showed reactivity to cytokeratin (CK) 7 and CK 20. Postoperatively, the patient was managed with intravenous antibiotics, analgesics, and supportive care. The patient was hemodynamically and vitally stable and was discharged with advice to follow up in the surgical oncology outpatient department after seven days.

## Discussion

Of all bladder cancers, approximately 0.5%-2.5% are primary adenocarcinomas of the urinary bladder, making it an uncommon and aggressive cancer [1,2]. This tumor is histologically different from the more prevalent transitional cell carcinoma and originates from the glandular epithelium within the bladder. Diagnosing and treating primary bladder adenocarcinoma might be difficult due to its aggressive nature and rarity. Although the precise cause of primary adenocarcinoma of the bladder is still unknown, a number of variables have been suggested. It is believed that a major contributing factor to the development of this malignancy is persistent irritation and inflammation of the bladder mucosa. Adenocarcinoma may develop from the metaplasia of the urothelium into glandular epithelium caused by diseases like schistosomiasis, persistent urinary tract infections, and bladder calculi. Moreover, bladder exstrophy and urachal remnants are linked to a higher risk of adenocarcinoma. Specifically, urachal remnant-derived bladder adenocarcinomas are more likely to develop at the bladder's dome and exhibit distinct biological characteristics and prognoses in contrast to non-urachal bladder adenocarcinomas. Primary bladder adenocarcinoma usually manifests as nonspecific symptoms like dysuria, hematuria, pelvic pain, and frequency of urination, which are also present in other kinds of bladder carcinomas. The lack of distinct symptoms frequently causes a delay in diagnosis, which worsens its prognosis. When screening individuals with chronic urinary symptoms, especially when traditional therapies are ineffective, it is crucial to consider adenocarcinoma in the differential diagnosis due to the nonspecific character of these symptoms. Because primary bladder adenocarcinoma is uncommon and has histological and clinical characteristics with other bladder diseases, diagnosis can be difficult. A crucial diagnostic technique is cystoscopy, which enables direct sight of the bladder mucosa and biopsy of lesions that appear suspicious. CT and magnetic resonance imaging scans are two imaging tests that can reveal more details regarding the location, size, and potential invasion of surrounding components of a tumor. Nevertheless, histological biopsy or surgical specimen analysis is necessary for a conclusive diagnosis. Histopathologically, malignant glandular cells forming tubules or papillary structures are the hallmark of primary bladder adenocarcinoma. The differentiation of primary bladder cancer from metastatic adenocarcinomas arising from other locations, like the colon or prostate, might be aided by immunohistochemical staining. Because primary adenocarcinoma of the bladder is an aggressive tumor with a propensity for early invasion and metastasis, treatment is complicated. Typically, a multimodal strategy is used for treatment. The primary treatment for localized adenocarcinoma is a radical cystectomy, which entails removing the bladder along with any surrounding tissues, frequently including pelvic lymph nodes. The best chance for long-term survival may be provided by radical cystectomy in situations where the tumor is contained and resectable. When surgical resection is not possible, or the disease is advanced, chemotherapy and radiation therapy may be utilized as adjuvant therapies. Research on the efficacy of radiation and chemotherapy for primary bladder adenocarcinoma is ongoing, with conflicting results documented in the literature. Research on targeted therapeutics for bladder adenocarcinoma is still in progress. Molecular profiling and identifying certain mutations may present opportunities for customized treatment plans in the future. Primary bladder adenocarcinoma usually has a poor prognosis with a significant risk of metastasis and recurrence. The delayed appearance and diagnosis are the main reasons for the considerably worse five-year survival rate when compared to other forms of bladder cancer. Although the overall prognosis is still uncertain, early identification and vigorous treatment are essential for improving results. This example brings up a number of crucial points that physicians should remember. When assessing patients with chronic urinary symptoms, a strong index of suspicion is required due to the vague symptoms and the possibility of misdiagnosis. A similar case of primary urinary bladder mucinous adenocarcinoma was described in 2021 by Karikalan et al. [9]. Primary adenocarcinoma is an uncommon and difficult tumor of the urinary bladder with a dismal prognosis, according to research by Nocks et al. [10] and Anderström et al. [11]. When making a differential diagnosis, clinicians should consider primary bladder adenocarcinoma, especially for individuals who have risk factors or unusual clinical presentations. The instance also emphasizes the value of a multidisciplinary treatment plan that includes radiologists, pathologists, urologists, and oncologists. Collaborative care is necessary to maximize diagnostic precision and customize treatment regimens for each patient. More investigation into the molecular biology and pathophysiology of primary bladder cancer is required to create tailored treatments and enhance patient outcomes. Multicenter studies and clinical trials are crucial to creating standardized treatment procedures and investigating cutting-edge therapy modalities.

## Conclusions

In conclusion, primary urinary bladder adenocarcinoma is an uncommon, difficult-to-treat cancer with a dismal prognosis. Improving patient outcomes requires early detection, precise diagnosis, and all-encompassing care. In order to improve our knowledge and approach to treating this aggressive malignancy, ongoing research and clinical collaboration are essential.

## References

[1] Bates AW, Baithun SI (2000). Secondary neoplasms of the bladder are histological mimics of nontransitional cell primary tumours: clinicopathological and histological features of 282 cases. Histopathology.

[2] Bardales RH, Pitman MB, Stanley MW, Korourian S, Suhrland MJ (1998). Urine cytology of primary and secondary urinary bladder adenocarcinoma. Cancer.

[3] Wang HL, Lu DW, Yerian LM, Alsikafi N, Steinberg G, Hart J, Yang XJ (2001). Immunohistochemical distinction between primary adenocarcinoma of the bladder and secondary colorectal adenocarcinoma. Am J Surg Pathol.

[4] Melicow MM (1955). Tumors of the urinary bladder: a clinico-pathological analysis of over 2500 specimens and biopsies. J Urol.

[5] Williamson SR, Lopez-Beltran A, Montironi R, Cheng L (2011). Glandular lesions of the urinary bladder: clinical significance and differential diagnosis. Histopathology.

[6] Young RH (2009). Tumor-like lesions of the urinary bladder. Mod Pathol.

[7] Smith AK, Hansel DE, Jones JS (2008). Role of cystitis cystica et glandularis and intestinal metaplasia in development of bladder carcinoma. Urology.

[8] Cheng L, Montironi R, Bostwick DG (1999). Villous adenoma of the urinary tract: a report of 23 cases, including 8 with coexistent adenocarcinoma. Am J Surg Pathol.

[9] Karikalan B, Pasupati T, George SM (2021). Primary mucinous adenocarcinoma of the urinary bladder: a rare entity. West Afr J Med.

[10] Nocks BN, Heney NM, Daly JJ (1983). Primary adenocarcinoma of urinary bladder. Urology.

[11] Anderström C, Johansson SL, von Schultz L (1983). Primary adenocarcinoma of the urinary bladder. A clinicopathologic and prognostic study. Cancer.

